# Editorial: Smelly Fumes: Volatile-Mediated Communication between Bacteria and Other Organisms

**DOI:** 10.3389/fmicb.2016.02031

**Published:** 2016-12-20

**Authors:** Laure Weisskopf, Choong-Min Ryu, Jos M. Raaijmakers, Paolina Garbeva

**Affiliations:** ^1^CHANGINS, Viticulture and Oenology, University of Applied Sciences and Arts Western SwitzerlandNyon, Switzerland; ^2^Molecular Phytobacteriology Laboratory, Infectious Disease Research Center, Korea Research Institute of Bioscience and BiotechnologyDaejeon, South Korea; ^3^Department of Microbial Ecology, Netherlands Institute of Ecology (NIOO-KNAW)Wageningen, Netherlands; ^4^Molecular Biotechnology, Institute of Biology, Leiden UniversityLeiden, Netherlands

**Keywords:** volatiles, natural functions, microorganisms, plants, interactions

Volatiles are small (<300 Da), smelly molecules emitted by all organisms. They have very diverse roles for the producing organism (e.g., as infochemicals or antimicrobial compounds) and fulfill important ecosystem functions. While the importance of plant volatiles has been recognized for more than 30 years, research on microbial volatiles attracted attention only in the last decades. This special issue focuses on several new findings and recent developments in the field of microbial (fungal and bacterial) volatiles, their biological functions and chemical identification, which are highlighted in this editorial.

## Natural functions of microbial volatiles

Already at the very start of this research field, it became apparent that several microbial volatiles can modulate plant growth and have both plant growth-promoting and disease-suppressing activities (Ryu et al., 2003, 2004; Bailly and Weisskopf, 2012; Li et al., 2016). In this special issue, two papers describe the role of fungal volatiles on plant growth and defense (Bitas et al.; Kottb et al.): Bitas and colleagues studied volatile-mediated signaling between fungi and plants using a nonpathogenic *Fusarium oxysporum* and *Arabidopsis thaliana* as model organisms. They showed that fungal volatiles can enhance root and shoot biomass production through an auxin-dependent mechanism (Bitas et al.). In contrast, *Trichoderma* volatiles did not induce growth promotion in *A. thaliana* but triggered enhanced expression of defense-related genes and accumulation of phytoalexins, suggesting that plants can discriminate between different types of microbial volatiles (i.e., between those produced by *Trichoderma* and those produced by *Fusarium* strains) and induce different responses (Kottb et al.). 6-pentyl-alpha-pyrone (6PP) was identified as the main volatile in the headspace of *Trichoderma* and exposure of *A. thaliana* to pure 6PP mimicked the effect of the whole blend with respect to the increased expression of defense-related genes involved in the salicylic acid- and ethylene-mediated pathways (Kottb et al.). In the study of Song et al., the treatment with the plant volatile 3-pentanol led to an increased expression of defense-related genes involved in both the salicylic acid and the jasmonic acid-mediated pathways in *A. thaliana*, which in turn triggered resistance to the bacterial leaf pathogen *Pseudomonas syringae* pv. tomato (Song et al.). Similar induction of genes involved in both plant immune systems was observed in *A. thaliana* upon exposure to bacterial volatiles, as reported by Sharifi and Ryu and reviewed by Liu and Zhang.

Beyond stimulation of the plant immune system, many papers in this special issue addressed the direct role of bacterial volatiles in disease protection, i.e., through direct inhibition of pathogens: *Streptomyces* strains isolated from disease-suppressive soils emitted volatiles that reduced the growth of *Rhizoctonia solani*, which was mediated, at least partly, by 2-methylpentanoate and 1,3,5-trichloro-2-methoxy benzene. Additionally, the same isolates also demonstrated volatile-mediated plant growth promotion of *A. thaliana* (Cordovez et al.). Along the same lines, six rhizobacteria isolated from common bean, able to protect bean plants from the common bacterial blight (CBB) causal agent, were evaluated *in vitro* for their potential antifungal effects toward different plant pathogenic fungi (Giorgio et al.). The six rhizobacteria caused strong volatile-mediated inhibition of mycelial growth of *Sclerotinia sclerotiorum*. Volatile-mediated effects on the target fungus were further investigated by electron microscopy, which revealed multifaceted effects of bacterial volatiles on the fungal cells, including alteration of membranes, mitochondria and endoplasmic reticulum (Giorgio et al.). Natural enemies of fungi might also be a source of antifungal volatiles, as shown by the work of Lo Cantore et al.. They investigated the effects of volatiles from *Pseudomonas tolaasii*, a major bacterial pathogen of mushrooms and observed volatile-mediated inhibition of mycelium growth of different basidiomycetes. These volatiles also affected plant growth negatively or positively depending on compound and dose (Lo Cantore et al.), highlighting the need for testing different concentrations within the biologically relevant range when assessing the bioactivity of volatiles. Sulfur compounds and 1-undecene detected, among other volatiles, in the blends of potato-associated *Pseudomonas* strains showed adverse effects on the oomycete pathogen *Phytophthora infestans*. In this work, small sulfur containing volatiles proved most efficient in inhibiting different life stages of the late blight pathogen *in vitro* and *in planta* (De Vrieze et al.). Volatile-mediated bacteria-fungal interactions have mainly focused on suppression of fungal pathogens. The reverse effect of fungal volatiles on bacteria, however, has been largely ignored. In this special issue, Schmidt et al. revealed that rhizosphere bacteria can distinguish between different fungi and oomycetes based on their volatile blends. Bacterial volatiles also affect other bacteria as reviewed in Audrain et al. and as highlighted in the work of Tyc et al., who showed that the production of volatiles such as indole was significantly affected by interspecific bacterial interactions.

## Volatile production and ecosystem functioning

The vast majority of studies on microbial volatiles performed to date have used strains growing in isolation on artificial media. However, little is known about volatile emission in an ecosystem context. Van Agtmaal et al. investigated the role of microbial volatiles in suppressiveness of soils against the oomycete pathogen *Pythium intermedium*. They observed that anaerobic soil disinfestation used to kill soil-borne pathogens caused significant changes in soil community composition and temporary reduction of volatile emission. Another study also performed in soil with synthetic communities reported that interspecific interactions have a strong effect on volatile production and that the slow-growing and low-abundant strains significantly affected the emission of volatiles by the whole microbial community. Moreover, this study revealed that volatiles emitted by strains with direct access to nutrients may activate the growth of distantly located dormant bacteria (Schulz-Bohm et al.).

## Volatile detection and identification

Microbial volatiles are chemically highly diverse (Schenkel et al.; Kanchiswamy et al.) as they derive from various biosynthetic pathways. The technical developments in mass spectrometry that have been made in the recent years have led to the improvement of volatile compound detection and identification. However, the main challenge in volatile metabolomics, also referred to as “volatolomics,” is the ability to identify and quantify the blends of emitted volatiles produced *in situ*. These blends are usually highly complex and often contain a significant proportion of yet unidentified compounds. This makes the identification of biologically relevant volatiles a challenging task. This special issue contains several reports where detailed workflows for volatile analysis are presented (Tyc et al.; Schmidt et al.; Cordovez et al.), including the application of freely available software packages (such as MetAlign, mzMine, MetaboAnalyst, AMDIS) suitable for metabolomic analysis of volatile compounds. One additional challenge of working with microbial volatiles is the experimental design that allow high through-put analysis of the biological effects they have on target organisms while excluding effects mediated by non-volatile compounds (Cernava et al.).

## Outlook

The prominent role of microbial volatiles in the interaction with eukaryotes and in particular with plants has become more evident in the past decade. In contrast to above-ground interactions, exchange of volatile signals in the rhizosphere is largely understudied, mainly because of the physical-chemical and (micro)biological complexity of the root-soil interface. New methods such as those described by Kai et al. will help to tackle this challenge and will significantly improve our understanding of the biological significance of volatile-mediated plant-microbe interactions, both below- and above-ground. Ultimately, this knowledge can be translated into innovative strategies for a more sustainable crop production by applying volatiles as alternatives to deleterious pesticides or as environmentally friendly gaseous biofertilizers (Kanchiswamy et al.). For example, dimethyl disulfide, a volatile frequently emitted by many bacteria has been used in the recent years as the novel soil fumigant PALADIN® against nematodes and soil-borne pathogens. The research on the application of microbial volatiles in agriculture is still in its infancy. Further studies are needed to harness the potential of volatiles and to bring the knowledge from laboratory to field conditions.

## Author contributions

All authors LW, C-MR, JR, and PG, contributed in writing this Editorial article for the research topic “Smelly fumes: volatile-mediated communication between bacteria and other organisms”.

### Conflict of interest statement

The authors declare that the research was conducted in the absence of any commercial or financial relationships that could be construed as a potential conflict of interest.
